# Ambient Temperature Shapes Skeletal Muscle Growth and Fiber-Type Plasticity in Mice

**DOI:** 10.3390/cells15080685

**Published:** 2026-04-13

**Authors:** Yajie Dong, Wen Sun, Yanjun Dong, Yiran Xu, Linli Xue, Jiayin Lu, Yi Yan, Xiaomao Luo, Haidong Wang, Juan Wang

**Affiliations:** 1 College of Veterinary Medicine, Shanxi Agricultural University, Jinzhong 030801, China; b20221055@stu.sxau.edu.cn (Y.D.); 18634747958@163.com (Y.D.); 20233867@stu.sxau.edu.cn (Y.X.); xuelinli@sxau.edu.cn (L.X.); lujiayin2013@163.com (J.L.); yanyi@sxau.edu.cn (Y.Y.); xmluo@sxau.edu.cn (X.L.); 2 Department of Nephrology, Shanghai General Hospital, Shanghai Jiao Tong University School of Medicine, Shanghai 200080, China; sunwensj@sjtu.edu.cn

**Keywords:** ambient temperature, skeletal muscle remodeling, fiber type plasticity, intestinal morphology

## Abstract

**Highlights:**

**What are the main findings?**

- Ambient temperature shapes skeletal muscle plasticity in mice.
- 20 °C is associated with relatively preserved muscle function and altered myogenic markers.

**What is the implication of the main finding?**

- Temperature-dependent MyHC responses are non-linear and vary across conditions.
- Thermal extremes impaired muscle function and altered intestinal morphology.

**Abstract:**

Skeletal muscle development and physiological homeostasis are profoundly influenced by environmental cues. Among these factors, ambient temperature represents a critical determinant of growth performance and metabolic adaptation in mammals. However, the effects of different ambient temperature ranges on skeletal muscle characteristics and on responses across multiple visceral tissues remain poorly understood. In this study, five ambient temperature conditions (16 °C, 20 °C, 24 °C, 28 °C, and 32 °C) were established to investigate their physiological impacts in a mouse model. Our results demonstrate that ambient temperature markedly influences growth performance and skeletal muscle phenotype. Notably, mice housed at 20 °C showed relatively preserved grip strength and a shift in myofiber cross-sectional area distribution, although these findings did not consistently indicate superior skeletal muscle development across all indices. Further analysis revealed that ambient temperature significantly modulated the expression profiles of myosin heavy chain (MyHC) isoforms in skeletal muscle. Specifically, cold exposure was associated with an upregulation of the slow-twitch-related MyHC I, whereas heat stress correlated with an elevation of the fast-twitch-related MyHC IIb. Functional assessments indicated that exposure to colder or hotter conditions was associated with impaired muscle performance, as reflected by reduced grip strength at 16 °C, 28 °C, and 32 °C, and decreased endurance capacity at 28 °C and 32 °C. Histological analyses of major visceral organs revealed no obvious structural alterations in the heart, liver, spleen, lung, or kidney across temperature conditions. However, exposure to thermal extremes (16 °C and 32 °C) significantly reduced intestinal villus height, suggesting compromised intestinal integrity under temperature stress. Collectively, these findings indicate that ambient temperature is associated with multi-tissue changes in skeletal muscle characteristics, functional performance, and intestinal morphology. This study provides new insights into how environmental temperature modulates tissue adaptation and physiological homeostasis in mammals.

## 1. Introduction

Ambient temperature is a fundamental environmental factor that profoundly influences the survival, physiology, and health of organisms [1]. In mammals, temperature plays a critical role in regulating metabolic rate, energy balance, and physiological activities, thereby shaping growth and tissue development [2]. Skeletal muscle, the largest metabolic organ in the body, is essential for locomotion and whole-body energy homeostasis [3]. Meanwhile, visceral organs—including the digestive, immune, respiratory, and circulatory systems—are indispensable for nutrient absorption and physiological stability. However, although temperature is known to affect metabolic adaptation, the systemic effects of different ambient temperature ranges on skeletal muscle development and the coordinated responses of visceral organs remain poorly understood. Clarifying these responses will provide important insights into environmental adaptation and physiological homeostasis in mammals.

Temperature fluctuations serve as potent stressors [4]. In many geographical regions, prolonged exposure to cold environments during winter disrupts physiological equilibrium, leading to reduced production performance, decreased immune function, disease, and even death of animals [5,6]. Studies in livestock have shown that cold stress can severely affect productivity and behavior [7]. Conversely, with global warming, elevated temperatures pose a growing threat by increasing thermal load. Heat stress disrupts normal metabolism, decreases feed intake, and impairs growth performance and disease resistance [8,9]. For instance, high ambient temperatures have been shown to reduce muscle development and metabolic efficiency in lambs [10]. These findings highlight the sensitivity of mammalian growth to thermal variations.

Skeletal muscle exhibits remarkable plasticity in response to external stimuli [11]. Its development mainly involves the expression of Pax7 and its downstream regulation by key molecules such as myogenic regulatory factors (MRFs) [12], including MyoD, Myf5, MyoG, and MRF4 [13,14]. Crucially, this process is modulated by environmental conditions [15]. For example, low temperature exposure has been reported to induce adverse structural and metabolic changes in skeletal muscle, potentially affecting muscle quality and function [16,17,18]. Transcriptomic analysis demonstrated that prolonged heat stress perturbed the metabolic activity and structural integrity of muscle tissue [19], leading to inhibited protein synthesis and enhanced catabolism [20]. A key aspect of muscle adaptation involves the remodeling of muscle fiber types. While animal myofiber number is determined in the embryonic period, fiber type and composition remain plastic postnatally, allowing adaptation to environmental demands. Research indicates that low temperatures can induce a shift toward oxidative, type I fibers [21]. In contrast, continuous high temperatures may decrease type I content and promote a transition toward glycolytic, type II fibers [22]. This adaptive remodeling process of myofiber type is essential for alleviating skeletal muscle fatigue, improving tissue functional quality, and maintaining body energy homeostasis. The organ index can, to some extent, reflect the physiological functions and development status of an animal’s body. Meanwhile, the internal organs are important sites for the organism to absorb and utilize various nutrients. Their development affects the health of the animals. Only when the internal organs develop fully can the effective growth of tissues such as muscles be ensured [23].

In the present study, we systematically evaluated the effects of five distinct ambient temperatures on growth performance in mice, aiming to explore the differential impacts of temperature on their overall physiological status. Further analysis of skeletal muscle growth and development revealed that ambient temperature variations affect myofiber development and shift the expression profile of skeletal muscle MyHC isoforms, potentially altering contractile properties. Additionally, we evaluated the morphological response of visceral organs, particularly the intestine, to characterize cross-tissue changes involving intestinal morphology and skeletal muscle traits. These findings provide multi-dimensional theoretical insights into how organisms adapt to environmental thermal challenges.

## 2. Materials and Methods

### 2.1. Mice

Male C57BL/6 mice (4 weeks old) were procured from the Laboratory Animal Center, Shanxi Provincial People’s Hospital. They were certified as specific pathogen-free (SPF) by the China Institute of Food and Drug Control. The mice were healthy, wild-type, and unmodified, with no prior procedures. All animal experimental procedures received formal approval from the Institutional Animal Care and Use Committee (IACUC) of Shanxi Agricultural University (Approval No: SXAU-EAW-2023M.DY.003009159; Date: 8 March 2023).

### 2.2. Animal Treatment and Tissue Collection

C57BL/6J mice were acclimated for 1 week at 24 °C with free access to standard chow and water, then randomly divided into five groups (6 mice per group). Mice in the experimental cohort were exposed to 4 h of daily conditioning in an artificial climate chamber (Shanghai Chuanhong Experimental Instrument Co., Ltd., Shanghai, China), where all environmental variables matched the control group except for ambient temperature. The environmental temperatures are 16 °C, 20 °C, 24 °C, 28 °C, 32 °C. After acclimation, the mice were raised in artificial climate boxes (24 h per day) for 4 weeks. Rectal temperature was measured at 9 a.m., 3 p.m., and 9 p.m. At 3 p.m., mass and food intake were measured, and normal feeding management was performed in all groups. At 3 p.m. on day 30 of cold stimulation, the treatment was finished for all groups. Mice were anesthetized via intraperitoneal injection of 3% sodium pentobarbital (Sigma-Aldrich, St. Louis, MO, USA). Upon the onset of anesthesia, blood samples were collected. Subsequently, the mice were euthanized by cervical dislocation, and visceral organs, along with gastrocnemius muscle tissues, were immediately dissected and harvested. To ensure consistency in sampling, specific anatomical sites were standardized: the left ventricle was sampled for the heart, the inferior lobe of the left lung for the lung, and the mid-segment of the duodenum (2–3 cm distal to the pylorus) for the intestine.

### 2.3. Strength and Exercise Endurance Measurements

Muscle force was measured with a grip strength meter (Jiangsu Saiangsi Biotechnology Co., Ltd., Nantong, China): mice grasped the device’s horizontal metal grid with all limbs and were pulled backward for three trials. Treadmill endurance testing (Jiangsu Saiangsi Biotechnology Co., Ltd., Nantong, China) followed a 2-day acclimation period. Mice ran on a 5% inclined treadmill, with speed increasing by 2 cm/s every 5 min. Exhaustion was determined when hindlimb contact with the electric grid lasted >15 s, and the total time/distance was auto-recorded.

### 2.4. Glucose Tolerance Test (GTT) and Insulin Tolerance Test (ITT)

GTT and ITT were conducted using a glucometer (Sinocare Inc., Changsha, China) for glucose measurement. Intraperitoneal injections of glucose (1 g/kg) after overnight fasting (GTT) and insulin (1 U/kg) after 4-h fasting (ITT) were administered, correspondingly. Blood glucose levels were assessed via tail vein sampling at 0, 15, 30, 60, 90, and 120 min post-injection.

### 2.5. Serum Assay

Serum TC, TG, lipase, and glucose were detected via an automatic blood biochemistry analyzer (Biobase Biotechnology Co., Ltd., Jinan, China).

### 2.6. HE Staining

Muscle tissues were dehydrated in 30% glucose solution, frozen in liquid nitrogen, sectioned using a freezing microtome (Leica Microsystems (Shanghai) Co., Ltd., Shanghai, China), and stained with hematoxylin and eosin (HE) (Solarbio Life Science Co., Ltd., Beijing, China). Visceral organs were fixed in 4% paraformaldehyde (Solarbio Life Science Co., Ltd., Beijing, China) for 24 h, dehydrated through a graded ethanol series, cleared in xylene, embedded in paraffin, sectioned at 5 μm, and stained with HE. All sections were observed under a light microscope (Nikon Instruments (Shanghai) Co., Ltd., Shanghai, China).

### 2.7. Oil Red O Staining

After staining with Oil Red O (Solarbio Life Science Co., Ltd., Beijing, China), 60% isopropanol (Tianjin Hengxing Chemical Reagent Manufacturing Co., Ltd., Tianjin, China), hematoxylin (Solarbio Life Science Co., Ltd., Beijing, China) staining, differentiated with 1% hydrochloric acid, rinsed in tap water for 10 min, and glycerol gelatin (Solarbio Life Science Co., Ltd., Beijing, China) sealed under the microscope. Following staining, ImageJ 1.5 (National Institutes of Health, Bethesda, MD, USA) was used to quantify lipid droplet area. The percentage of lipid droplet area relative to the total field area was calculated and used as an index for evaluating intramuscular fat content.

### 2.8. RNA Extraction and RT-PCR

RNA extraction was conducted with TRIzol (Takara Bio Inc., Dalian, China) per the manufacturer’s instructions. cDNA synthesis utilized HiScript III RT SuperMix for qPCR (+gDNA wiper) (Vazyme Biotech Co., Ltd., Nanjing, China), and gene-specific primers were designed in Primer Premier 5.0 (Premier Biosoft, Palo Alto, CA, USA) based on cDNA sequences (see Table 1 for primer sequences). RT-qPCR reactions were run on a QuantStudio™ 5 System (Thermo Fisher Scientific, Waltham, MA, USA) with MonAmp SYBR Green qPCR Mix (Monad Biotechnologies, Suzhou, China). Four valid replicates were included per sample; relative expression was calculated using the 2^−ΔΔCt^ method, and differences were considered significant at *p* < 0.05.

### 2.9. Western Blot

The protein fractions were isolated with RIPA buffer (Beyotime Biotechnology, Shanghai, China) containing protease inhibitors (Servicebio Technology Co., Ltd., Wuhan, China). Protein samples (25 μg) were separated by SDS-PAGE, and electrophoresis was performed on ice, initially at 80 V for 30 min until the marker bands were sufficiently resolved, after which the voltage was increased to 120 V. The run was terminated when the bromophenol blue front reached the bottom of the gel. A 5% stacking gel was used, and the concentration of the resolving gel was determined based on the molecular weight of the target protein. The membranes were transferred to PVDF membranes (Millipore Sigma, Burlington, MA, USA), blocked for 1 h at 37 °C, and incubated with the relevant primary antibody overnight at 4 °C. The primary antibodies included MyoD (Abcam, Cambridge, UK; ab16148, Mouse, 1: 1000), MyoG (Santa Cruz Biotechnology, Dallas, TX, USA; sc-12732, Mouse, 1: 500), MSTN (proteintech, Wuhan, China; 19142-1-AP, Rabbit, 1: 1000), FBXO32 (ABclonal, Wuhan, China; A3193, Rabbit, 1: 2000), TRIM63 (Santa Cruz Biotechnology, Dallas, TX, USA; sc-398608, Mouse, 1: 1000), MyHC IIx (proteintech, Wuhan, China; 67299-I-Ig, Mouse, 1: 1000), MyHC IIb (proteintech, Wuhan, China; 20140-1-AP, Rabbit, 1: 1000), MyHC I (proteintech, Wuhan, China; 22280-1-AP, Rabbit, 1: 1000), GAPDH (proteintech, Wuhan, China; 10494-1-AP, 1: 10,000). After washing with TBST, membranes were incubated with HRP-conjugated IgG secondary antibodies at 37 °C for 1 h. Protein band visualization was achieved with ECL chemiluminescence reagent (Abbkine Scientific Co., Ltd., Wuhan, China), and band intensity was assessed via ImageJ 1.5 software (National Institutes of Health, Bethesda, MD, USA).

### 2.10. Statistical Analysis

All data are expressed as the mean ± SEM. Comparisons among multiple groups were performed using One-way ANOVA, followed by Tukey’s post hoc multiple comparisons test when significant differences were detected. A value of *p* < 0.05 was considered statistically significant. All statistical analyses were conducted using GraphPad Prism 9.0 software (GraphPad Software, San Diego, CA, USA).

## 3. Result

### 3.1. Ambient Temperature Modulates Systemic Metabolism and Growth Trajectories

**Figure 1:**
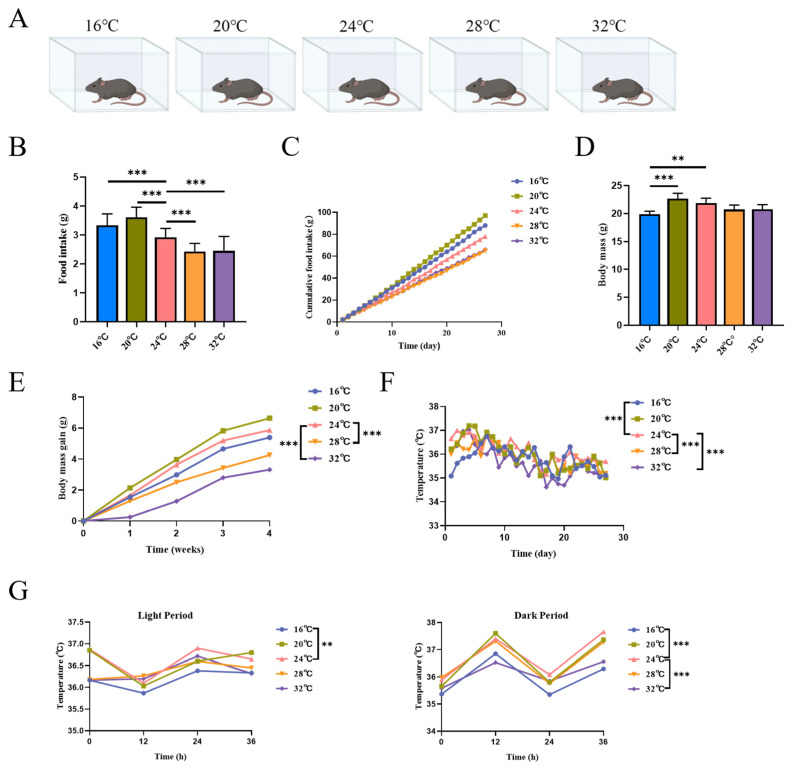
Effects of different environmental temperatures on the growth and development of mice. (**A**) Experimental scheme, male C57BL/6J mice were kept in artificial climatic chambers at 16 °C, 20 °C, 24 °C, 28 °C, and 32 °C for 4 weeks (*n* = 6). (**B**) Food intake of five groups of mice (*n* = 6). (**C**) Cumulative food intake (*n* = 6). (**D**) Final mass of the mice (*n* = 6). (**E**) Daily body mass gain of the five groups of mice (*n* = 6). (**F**) Core temperature of mice in Group 5, raised in climatic chambers for 4 weeks (*n* = 6). (**G**) Diurnal body temperature of the five groups of mice at the beginning and end of the period (*n* = 6). Data were presented as mean ± SEM. **, *p* < 0.01; and ***, *p* < 0.001.

Environmental temperature exerts a profound influence on the overall state of the mammals, including physical activity and metabolism. Male C57BL/6J mice were fed in artificial climate chambers with different temperatures (16 °C, 20 °C, 24 °C, 28 °C, 32 °C) for 4 weeks (Figure 1A). As expected, food intake was significantly higher in the low-temperature environments (16 °C and 20 °C) than in the high-temperature environments (28 °C and 32 °C) (Figure 1B), a trend further corroborated by cumulative food intake data (Figure 1C). This reflects the adaptive metabolic cost of thermoregulation. Consequently, terminal body mass at 16 °C was significantly lower than in the thermoneutral control group (24 °C) (Figure 1D). The body masses of the mice all showed an increasing trend with increasing day age, and body mass gain in the 16 °C, 20 °C, 24 °C, and 28 °C murine groups was rapid initially and slowed in the later stage; Mice grew slowly at 32 °C. However, body mass gain was greater in the 20 °C group than in the 16 °C group. The mass gain of mice in the 28 °C and 32 °C groups was significantly lower relative to the 24 °C control group (Figure 1E). Core body temperature analysis revealed that 20 °C did not significantly alter thermal homeostasis compared to controls, whereas deviations to 16 °C, 28 °C, and 32 °C resulted in significant hypothermia or hyperthermia-induced adaptations (Figure 1F). Tracking of diurnal rectal temperature dynamics (Figure 1G) further highlighted that cold exposure (16 °C) significantly dampened diurnal thermal rhythms initially, indicative of metabolic stress.

**Figure 2:**
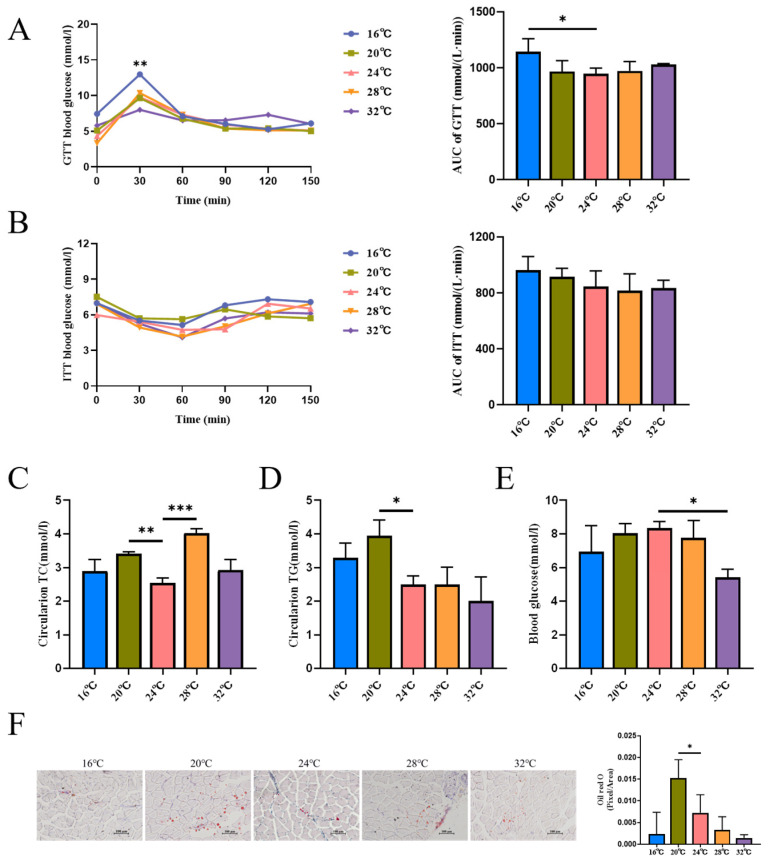
Effects of different environmental temperatures on the growth and development of mice. (**A**) Intraperitoneal GTT and the area under the GTT curve (*n* = 6). (**B**) ITT and its area under the curve (*n* = 6). (**C**–**E**) Blood biochemical indices in five groups of mice (*n* = 3). (**C**) Total cholesterol. (**D**) Triglycerides. (**E**) Blood glucose. (**F**) Oil red O staining of gastrocnemius muscle in five murine experimental groups and the corresponding quantitative analysis results (scale bar: 100 µm). Data were presented as mean ± SEM. *, *p* < 0.05; **, *p* < 0.01; and ***, *p* < 0.001.

Moreover, GTT and ITT showed (Figure 2A,B) that blood glucose increased at 16 °C, potentially indicating altered glucose utilization, although insulin sensitivity remained largely unaffected across groups. To further assess their status, we measured blood biochemical levels after 4 weeks (Figure 2C–E). The blood biochemical indices can, to a certain extent, reflect the animal’s nutrient metabolism and health status and serve as a reference for subsequent studies. In this experiment, blood glucose decreased at 32 °C, whereas there was no notable change in the other groups. Total cholesterol increased significantly at 20 °C and 28 °C, and triglyceride increased at 20 °C. Total cholesterol and triglyceride levels did not differ notably between the 16 °C and 32 °C groups. It is inferred that there is a significant difference in lipid metabolism at 20 °C. Oil red O staining can further determine the deposition of triglycerides in muscle tissue. The intermuscular fat of mouse skeletal muscle was stained by oil red O dye solution (Figure 2F), and it was observed that mice in the 20 °C group exhibited significantly greater intermuscular fat accumulation compared to the other groups. These findings further suggest that varying ambient temperatures may directly influence lipid metabolism within skeletal muscle tissue, thereby modulating the metabolic characteristics of the skeletal muscle.

### 3.2. Ambient Temperature Modulates Myogenic Potential

**Figure 3:**
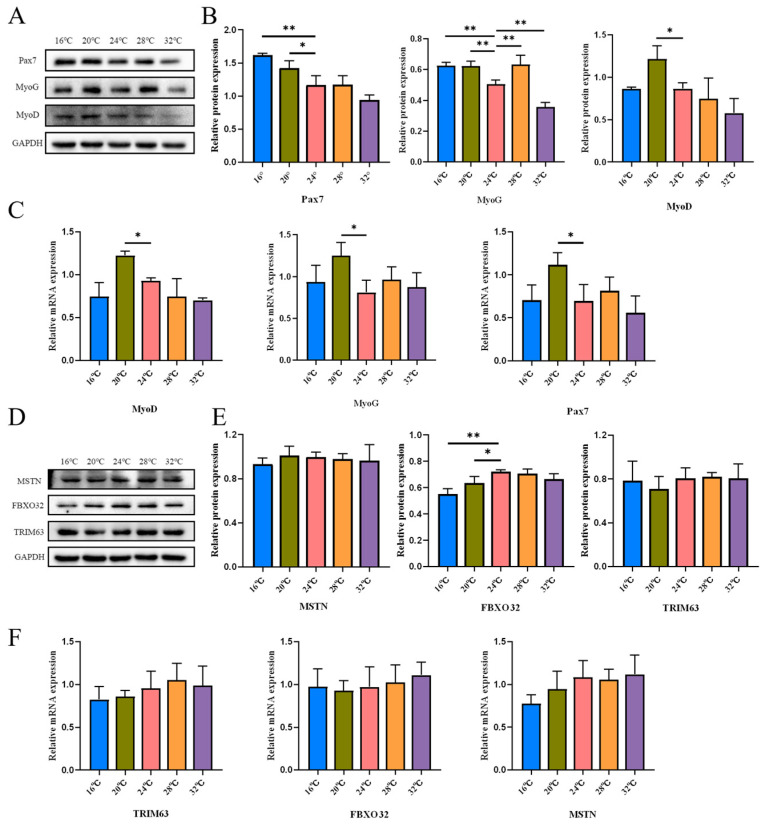
Effects of different environmental temperatures on myofiber development. (**A**) Immunoblot strip chart of muscle growth and development-related Pax7, MyoD, and MyoG in gastrocnemius muscle (*n* = 3). (**B**) Pax7, MyoD, and MyoG Protein expression in gastrocnemius muscle (*n* = 3). (**C**) Pax7, MyoD, MyoG mRNA Levels in Gastrocnemius Muscle (*n* = 3). (**D**) Immunoblots of muscular atrophy MSTN, FBXO32, and TRIM63 in Gastrocnemius (*n* = 3). (**E**) MSTN, FBXO32, and TRIM63 Protein Expression Profiles in Murine Gastrocnemius Muscle (*n* = 3). (**F**) mRNA Expression Analysis of MSTN, FBXO32, and TRIM63 in Gastrocnemius Muscle Tissues (*n* = 3). Data were presented as mean ± SEM. *, *p* < 0.05; and **, *p* < 0.01.

It has been shown that SCs contribute not only to postnatal myofiber formation but also to skeletal muscle development [24]. To determine if ambient temperature regulates myogenesis, we analyzed the expression of muscle development-related genes under distinct temperature regimes (Figure 3A–C). At the transcriptional level, Pax7 expression was significantly upregulated at 20 °C (Figure 3C). Consistent with this, Western blot analysis revealed notably enhanced Pax7 protein abundance at both 16 °C and 20 °C, suggesting that cooler environments may stimulate SC pool maintenance or activation. Furthermore, downstream differentiation markers, MyoD and MyoG, showed upregulated mRNA and protein expression at 20 °C. Notably, MyoG protein levels were elevated across the cooler spectrum (16–20 °C) but suppressed at 32 °C (Figure 3B,C), indicating that heat stress may impair differentiation capacity. In addition, Western blot and qPCR demonstrated that FBXO32 protein expression was significantly reduced after lowering the temperature to 16 °C and 20 °C, while the mRNA did not change (Figure 3D–F). The expression of MSTN and TRIM63 did not change at different environmental temperatures. Results indicated that 20 °C suppressed the protein expression of the muscle atrophy factor FBXO32, whereas it had no significant effect on MSTN or TRIM63. In summary, ambient temperature altered the expression of myogenesis-related markers. The 20 °C condition was associated with increased Pax7 and myogenic differentiation markers, although similar changes in some markers were also observed at 16 °C, indicating that the molecular response was not unique to 20 °C.

### 3.3. Thermal Stress Drives Adaptive Remodeling of Skeletal Muscle MyHC Isoforms

**Figure 4:**
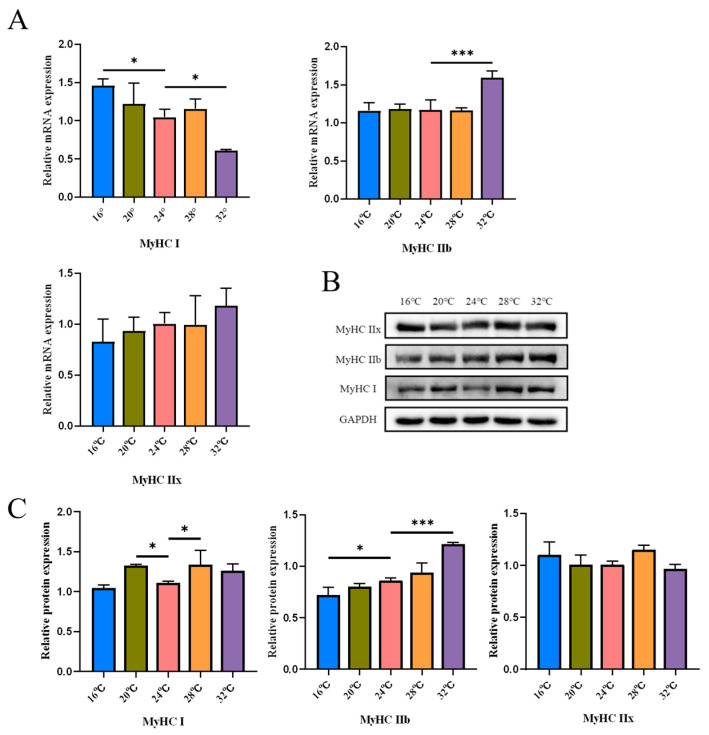
Ambient temperature drives skeletal muscle fiber type transformation. (**A**) mRNA expression of MyHC I, MyHC IIb, and MyHC IIx in the gastrocnemius muscle, assessed via qPCR (*n* = 3). (**B**) Immunoblotting strip chart of MyHC I, MyHC IIb, and MyHC IIx (*n* = 3). (**C**) Western blot of MyHC I, IIb, and IIx protein levels in the gastrocnemius muscle (*n* = 3). Data were presented as mean ± SEM. *, *p* < 0.05; and ***, *p* < 0.001.

Skeletal muscle exhibits high plasticity, allowing it to adjust its metabolic and functional properties in response to environmental demands [25,26]. In this study, we investigated the effects of different temperatures on the expression of MyHC isoforms in the gastrocnemius muscle to evaluate the impact of thermal stimuli on the molecular phenotype of muscle fibers. In the gastrocnemius muscle, compared with the 24 °C control group, the 16 °C group showed a significant increase in MyHC I mRNA expression, while MyHC IIb mRNA levels showed no significant difference. The 20 °C group exhibited no significant changes in either MyHC I or MyHC IIb mRNA expression (Figure 4A). Western blot analysis revealed that, compared to the 24 °C group, MyHC IIb protein expression was decreased in the 16 °C group, whereas MyHC I protein was significantly upregulated in the 20 °C group (Figure 4B,C). These results suggest that lower temperature conditions induce changes in the MyHC isoform expression pattern in the gastrocnemius muscle. However, the response was not uniform across temperatures and did not support a simple linear shift toward a slow-twitch phenotype. At 28 °C, there were no significant differences in MyHC I and MyHC IIb mRNA expression levels, while MyHC I protein expression was significantly increased (Figure 4A–C). At 32 °C, MyHC IIb mRNA was significantly elevated and MyHC I mRNA was significantly reduced; Western blotting confirmed an increase in MyHC IIb protein expression (Figure 4A–C). Thus, the response of MyHC isoforms to ambient temperature was non-linear and temperature-specific. In particular, the increase in MyHC I protein at 28 °C suggests that regulation near thermoneutrality may differ from the responses observed under colder or hotter conditions. Overall, ambient temperature induced distinct remodeling of skeletal muscle MyHC isoform expression rather than a simple cold-to-slow or heat-to-fast transition.

### 3.4. Temperature-Associated Remodeling Is Accompanied by Changes in Muscle Function

**Figure 5:**
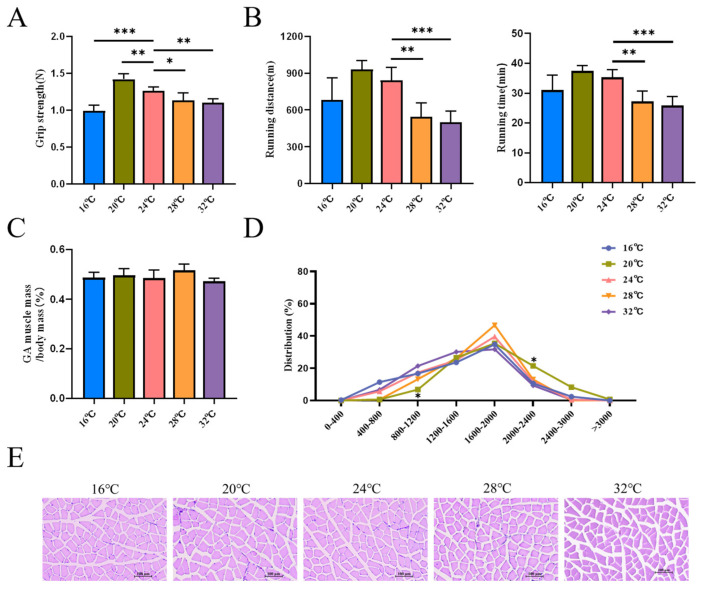
Temperature-induced muscle remodeling leads to distinct contractile functional changes. (**A**) Muscle grip strength test in five groups of mice (*n* = 6). (**B**) Endurance exercise test, time and distance to exhaustion measured (*n* = 6). (**C**) Gastrocnemius index (*n* = 6). (**D**) Frequency histograms of the cross-sectional area of gastrocnemius myofiber in five groups of mice (*n* = 3). (**E**) HE staining analysis of gastrocnemius muscle tissue (scale bar: 100 µm). Data were presented as mean ± SEM. *, *p* < 0.05; **, *p* < 0.01; and ***, *p* < 0.001.

To determine the functional consequences of these molecular and histological changes, we assessed muscle performance. Grip strength was significantly compromised in mice exposed to thermal deviations (16 °C, 28 °C, and 32 °C) (Figure 5A), indicating reduced maximal force production. Furthermore, treadmill endurance tests revealed impaired locomotor capacity (reduced time and distance) under heat stress (28 °C and 32 °C) (Figure 5B). Conversely, mice housed at 20 °C exhibited enhanced grip strength, suggesting improved functional output. Morphometric analysis of the gastrocnemius muscle revealed that, while the muscle-to-body mass ratio remained constant across groups (Figure 5C), the myofiber size distribution was significantly altered (Figure 5D). The distribution ratio of small myofiber (800–1200 µm^2^) was decreased, and the distribution ratio of large myofiber (2000–2400 µm^2^) was increased at 20 °C (Figure 5D,E). The distribution ratio of small myofibers increased at 16 °C and 32 °C, but the difference was not significant. These findings indicate that ambient temperature was associated with remodeling of myofiber cross-sectional area distribution rather than changes in overall muscle mass. In contrast, colder and hotter conditions were accompanied by impaired muscle performance.

### 3.5. Thermal Extremes Compromise Intestinal Morphology Despite Stable Organ Indices

Systemic adaptation to temperature also involves visceral organs. As presented in Table 2, organ coefficients for the heart, liver, spleen, lung, and kidney did not differ significantly across all experimental groups relative to the normothermic control group (24 °C). These results indicated that ambient temperature variations within the tested range did not interfere with the normal growth and development of visceral organs in mice, which aligns with the inherent ability of animals to regulate visceral organ development in accordance with the growth status of the whole organism.

**Figure 6:**
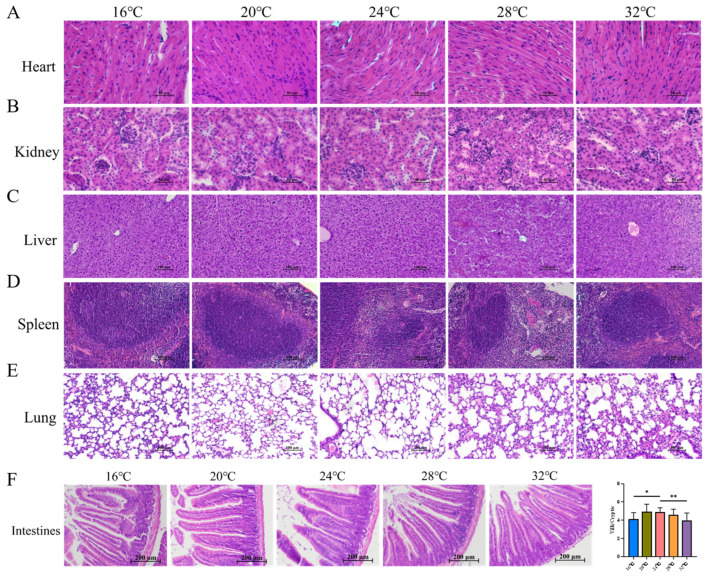
Histological analysis of the heart, liver, spleen, lung, kidney, and intestine in mice under different ambient temperatures. (**A**,**B**) Representative HE staining images of the heart (**A**) and kidney (**B**) in mice under different temperature conditions (Scale bar: 50 µm). (**C**–**E**) Representative H&E staining images of the liver (**C**), spleen (**D**), and lung (**E**) in mice under different temperature conditions (Scale bar: 100 µm). (**F**) Representative HE staining images of the intestine in mice under different temperature conditions (Scale bar: 200 µm) and the villus height-to-crypt depth ratio of the intestine (*n* = 6). Data were presented as mean ± SEM. *, *p* < 0.05; and **, *p* < 0.01.

However, histological evaluation revealed tissue-specific vulnerabilities. In cardiac tissue (Figure 6A), sections from the 16 °C group exhibited somewhat irregular arrangement of myocardial fibers, with occasional widening of the interstitial spaces. The 20 °C and 24 °C groups maintained relatively intact fiber architecture. In the 28 °C and 32 °C groups, mild undulation of the myocardial fibers was observed in some sections. Renal, hepatic, and splenic tissues remained largely unaffected, exhibiting normal morphology across groups, although mild congestion was noted in the liver at extreme temperatures (Figure 6B–D). Lung tissue (Figure 6E) exhibited signs of structural compromise under stress: 16 °C and 28 °C groups showed thickened alveolar walls and partial collapse in some lung tissue sections. Meanwhile, the 32 °C group displayed inflammatory cell infiltration and hemorrhage, indicative of heat-induced pulmonary injury. Critically, intestinal morphology was significantly impacted. While villus-to-crypt ratios were preserved at 20 °C and 24 °C, this ratio was markedly reduced in both cold (16 °C) and heat (32 °C) stress groups (Figure 6F). This villus atrophy suggests that thermal extremes compromise intestinal absorptive surface area and barrier integrity. In summary, while 20 °C supports optimal visceral health, 16 °C and 32 °C impose structural stress on the heart, lungs, and intestine.

## 4. Discussion

Ambient temperature acts as a pervasive environmental selector, shaping the physiological landscape of endotherms through complex regulatory networks. While optimal thermal conditions facilitate homeostasis and growth, thermal stress—whether cold or heat—imposes high metabolic costs and structural adaptations. In this study, we utilized a murine model to investigate physiological responses across multiple tissues to varying ambient temperatures. Our results indicate that deviations from standard housing temperatures elicit a series of alterations in skeletal muscle phenotypes, myogenesis-related molecular expression, and visceral tissue morphology. These findings suggest that ambient temperature exerts extensive effects on mammalian growth and tissue adaptation processes.

Herein, 24 °C was selected as the control temperature, primarily because it is a widely used standard for laboratory animal housing, facilitating comparisons with previous studies. However, the thermoneutral zone for mice is typically located between 28 °C and 30 °C, a range in which the organism can maintain thermal homeostasis without incurring additional metabolic costs for thermogenesis. Therefore, from a physiological perspective, the 24 °C condition and lower temperatures employed in this study effectively constituted varying degrees of cold stress for the mice. A fundamental principle of environmental physiology is the trade-off between thermoregulation and growth. When fattening pigs are raised in a high-temperature environment over an extended period, the organism’s metabolism changes, with spontaneous feed intake decreasing, average daily mass gain decreasing, and body heat gain decreasing, thereby maintaining body temperature at a normal level [27]. Previous studies have shown that chronic heat stress decreases feed intake and daily mass gain, and causes poor digestion and nutrient absorption in broilers [28]. Low ambient temperatures markedly alter metabolism and increase dietary intake in mammals [29]. The above studies show that different temperatures affect average daily mass gain, feed intake, and other traits of animals to varying degrees. In this study, average daily feed intake and daily gain in mice were notably reduced with increased temperature, consistent with prior investigations [30]. The reduction in food intake following a temperature increase is a protective and adaptive mechanism that offsets the increase in metabolic thermogenesis [31]. Elevated temperatures accelerate the activation of heat-dissipation mechanisms by decreasing food intake, thereby reducing energy demand. Meanwhile, increased temperature slows down gastrointestinal contraction and peristalsis; the rate of passage of chyme slows down, and the sense of satiety increases, which, in turn, reduces food intake. When the temperature decreases, animals need to adapt to the changing environment by shivering or increasing their feed intake [32]. In this study, final body mass and average daily feed intake in each group increased with decreasing temperature. Combined with daily mass gain in mice at different temperatures, as mice aged, all groups showed a trend of rapid early-stage body mass growth and slow late-stage growth. Therefore, 20 °C exhibited relatively more favorable characteristics for growth and muscle phenotype.

Glucose maintenance is important for animal survival [33]. After acute cold exposure, glucose uptake of brown fat in rats was enhanced, and glucose tolerance level was improved [34]. Herein, 16 °C elevated blood glucose, decreased glucose utilization, and elevated tolerance levels. Different temperatures did not seem to affect insulin tolerance. This observation is consistent with prior investigations [35], which found that housing temperature did not appear to affect insulin tolerance. To some extent, serum biochemical indices can reflect nutrient metabolism and animal health status. Therefore, this part of the experiment examines the effects of blood biochemical indices in mice at different temperatures, providing a basis for further investigation. The glucose level in the 32 °C group was lower than in the other groups. It suggests that animals have high energy requirements in high-temperature environments. However, with decreased feed intake, glucose intake is also reduced, resulting in low blood glucose levels. Triglycerides are important functional substances in the body and, together with cholesterol, are linked to lipid metabolism [36]. In this study, reducing the temperature to 20 °C increased TC and TG contents, suggesting a need for higher energy to maintain homeostasis. It indicates that adipose tissue catabolism was promoted at 20 °C. This finding was further corroborated by Oil Red O staining of intramuscular fat, which showed that its accumulation is regulated by ambient temperature, indicating that ambient temperature is a critical modulator of lipid metabolism.

Skeletal muscle plasticity is underpinned by the activity of satellite cells (SCs) [37]. Pax7, the first measurable marker of quiescent and activated SCs, is vital for SC viability and development [38]. MyoD functions mainly in myoblast formation, whereas MyoG—a core muscle development regulator—facilitates myoblast differentiation/fusion and induces the requisite target genes [39]. Loss of MyoG in skeletal muscle causes neonatal mortality in mice, demonstrating that MyoG is a key modulator of muscle growth and development [40]. In this work, the relative expression levels of MyoG, MyoD, and Pax7 genes and proteins were significantly increased at 20 °C. Notably, MyoG and Pax7 protein levels were also elevated in the 16 °C group, suggesting that these alterations are not exclusive to the 20 °C condition. Collectively, these findings indicate that 20 °C may favorably support skeletal muscle in maintaining a relatively optimal state of molecular adaptation. MSTN acts as a negative regulatory factor exerting important physiological effects, and its main role is to inhibit myoblast growth and differentiation [41]. TRIM63 and FBXO32 are muscle-specific ubiquitin ligases that accelerate protein degradation and muscle atrophy. This experiment found that different environmental temperatures did not significantly affect the expression of muscle atrophy-related genes, whereas reducing the temperature to 16 °C and 20 °C inhibited FBXO32 protein expression. The underlying causes of these divergent results warrant further investigation, and this alteration is likely associated with variations in myofiber type composition.

According to a previous report [22], compared with 28 °C, the proportion of type I muscle fibers in the skeletal muscle of large white pigs was significantly increased under 12 °C conditions, suggesting that a low-temperature environment may promote skeletal muscle adaptation towards a more oxidative metabolic phenotype. Previous studies have also indicated that chronic heat stress enhances glycolytic metabolism, reduces the proportion of type I muscle fibers, and shifts skeletal muscle towards a fast-twitch phenotype [42]. These studies provide a reference for understanding the regulation of skeletal muscle phenotype by temperature. Although the present study did not directly quantify muscle fiber type proportions, we observed changes in MyHC isoform expression that were generally consistent with previous reports. Specifically, under lower temperature conditions, MyHC I-related expression indices showed an upward trend. In contrast, MyHC IIb expression was significantly elevated at 32 °C, suggesting that different temperatures may induce distinct molecular expression characteristics related to muscle fibers in the gastrocnemius muscle. Importantly, the MyHC-related responses did not follow a simple linear pattern across ambient temperatures. Although some changes at 16 °C were partly consistent with a more oxidative profile, the significant increase in MyHC I protein at 28 °C indicates that regulation near thermoneutrality may differ from the responses observed under colder or hotter conditions. This finding suggests that temperature-related remodeling of muscle fiber phenotype is non-linear and temperature-specific. Although the underlying mechanism was not directly examined here, the increase in MyHC I protein at 28 °C may reflect altered energetic demand, contractile adaptation, or other regulatory processes under conditions close to thermoneutrality; however, this interpretation remains speculative. In addition, the dissociation observed between mRNA and protein expression should be interpreted cautiously. Such discrepancies may arise from post-transcriptional regulation, differences in translation efficiency, protein turnover, or temporal lag between transcriptional changes and protein accumulation. Accordingly, the present findings likely reflect a dynamic remodeling process rather than a simple transcriptionally driven fiber-type switch.

It has also been shown that the contractile capacity of skeletal muscle myofibers is tightly correlated with myofiber development [43]. Through further testing, we found that mice housed at 20 °C exhibited superior exercise performance in terms of grip strength and running time, suggesting that skeletal muscle functional status is relatively more favorable within this temperature range. However, no significant differences in muscle mass percentage were observed, which may be attributed to synchronous changes in total body weight and muscle weight under different temperature conditions. Interestingly, HE staining also showed that smaller myofibers (800–1200 µm^2^) decreased significantly and larger myofibers (2000–2400 µm^2^) increased significantly at 20 °C, indicating that ambient temperature can influence the distribution of muscle fiber cross-sectional area. At 16 °C and 32 °C, the proportion of small fibers increased, which may promote muscle atrophy and inhibit muscle function, affecting muscle development. It has been shown that acclimation to mild cold also causes a marked decrease in myofiber cross-sectional area [44]. Under heat stress, the cross-sectional area of gastrocnemius and soleus myofibers in rats was markedly diminished [45], which accorded with the findings of this investigation. This “structure-function” discordance highlights that while physiological plasticity aids survival, it may compromise physical performance.

Beyond the musculoskeletal system, environmental temperature exerted distinct toxicological effects on visceral organs. While gross organ indices remained stable—illustrating the organism’s prioritization of vital organ mass [23]—histological analysis revealed tissue-specific vulnerabilities. As the core organ of the circulatory system, the heart has been reported to exhibit pathological changes, including myocardial fiber rupture and cellular edema, in piglets subjected to acute cold exposure [46]. In the current study, myocardial fibers in mice of the 16 °C group showed structural disorder, dissolution, rupture, and widened interstitial spaces; the 20 °C group presented more tightly arranged myocardial fibers compared with the 24 °C group; a small quantity of wavy myocardial fibers was detected in the 28 °C and 32 °C groups. These findings indicate that different ambient temperatures may induce adaptive responses in myocardial tissue. The liver [47], spleen [48], and kidneys [49] are responsible for key physiological functions, including metabolism and detoxification, immune regulation, and waste excretion, respectively. No distinct changes in the histological morphology of the liver, spleen, and kidneys were observed among mice in different temperature groups, demonstrating that these organs developed normally within the temperature range tested in this study. As a vital organ for gas exchange, the lung is rich in capillaries and alveolar tissues, rendering it highly sensitive to ambient temperature stimuli and relatively weak in immune defense [50]. Previous studies have shown that cold exposure can lead to thickening of the alveolar interstitium in piglets [46]. In the present study, alveolar wall thickening was observed in the lung tissue of mice in the 16 °C, 28 °C, and 32 °C groups, suggesting that temperature conditions deviating from 24 °C may affect the morphological homeostasis of lung tissue and may be accompanied by some degree of inflammation-related response. Intestinal health directly modulates livestock and poultry growth, development, and nutrient utilization, and the villus height-to-crypt depth ratio is a validated core indicator of intestinal absorptive function [51]. Herein, this ratio was markedly reduced in mice from the 16 °C and 32 °C thermal conditions, indicating impaired intestinal digestive and absorptive capacity. This phenomenon may be attributed to gastrointestinal dysfunction induced by low temperature or to reduced gastrointestinal peristalsis caused by high-temperature adaptation. Concurrently, alterations in crypt depth suggest that the intestinal epithelial renewal process and tissue homeostasis may be influenced by ambient temperature [52], thereby further affecting the normal growth, development, and structural homeostasis of intestinal tissue.

Although our data suggest that ambient temperature may be involved in the regulation of skeletal muscle phenotypic adaptation, the key signaling networks underlying this process and its long-term adaptive characteristics warrant further elucidation. Moreover, due to the differences in physiological and metabolic characteristics between mice and large domestic animals, the conclusions of this study need further verification for their application in production practices. At the same time, this study has limitations, including a small sample size, the detection of only the gastrocnemius muscle, and a short exposure period. Therefore, the results should be regarded as preliminary observations. In the future, it is necessary to expand the sample size, include more muscle types, and extend the intervention period to verify and expand the current findings.

## 5. Conclusions

In conclusion, our study demonstrates that ambient temperature is a critical environmental factor influencing skeletal muscle phenotypes and physiological performance in mice. Compared with other thermal conditions, 20 °C exhibited relatively favorable characteristics in terms of skeletal muscle morphology, exercise performance, and myogenesis-related molecular indices. Conversely, conditions deviating from this temperature range induced alterations in the MyHC isoform expression pattern, accompanied by a decline in muscle performance. Furthermore, morphological alterations in intestinal tissue were observed under extreme temperature conditions, suggesting that thermal stress can impact the physiological status of multiple organs. Collectively, these findings reveal the systemic effects of ambient temperature on mammalian growth, skeletal muscle phenotypes, and organ homeostasis, providing valuable references for research on optimal growth-temperature mechanisms, the optimization of livestock production environments, and auxiliary interventions for muscle-related dysfunctions.

## Figures and Tables

**Table 1:** Related gene primer information table.

Primer Name	Primer Sequence 5′→3′
MyoD	F: ATGATGACCCGTGTTTCGACT R: CACCGCAGTAGGGAAGTGT
MyoG	F: ACAGCATCACGGTGGAGGATATGT R: CCCTGCTACAGAAGTGATGGCTTT
Pax7	F: GTTCGGGAAGAAAGAGGACGAC R: GGTTCTGATTCCACATCTGAGCC
MSTN	F: CAGACCCGTCAAGACTCCTAC R: CTGCCAAATACCAGTGCCT
FBXO32	F: TAGCATCGGTATGACTAAGT R: AGTCATATGGCAAGCATAC
TRIM63	F: TGATTCCTGATGGAAACGCT R: TCATTGGTGTTCTTCTTTACCCTC
MyHC I	F: CCATCTCTGACAACGCCTATC R: GGATGACCCTCTTAGTGTTGAC
MyHC IIx	F: ATGTTCCTGTGGATGGTCAC R: CTCGTTGGTGAAGTTGATGC
MyHC IIb	F: CTTGTCTGACTCAAGCCTGCC R: TCGCTCCTTTTCAGACTTCCG
β-actin	F: GAAGCTGTGCTATGTTGCTCTA R: CAATAGTGATGACCTGGCCGT

**Table 2:** Organ indices in mice under different ambient temperatures.

Organ Index	16 °C	20 °C	24 °C	28 °C	32 °C	*p*-Value
Heart	0.63 ± 0.073	0.63 ± 0.035	0.6 ± 0.04	0.56 ± 0.041	0.56 ± 0.074	0.147
Liver	6.22 ± 0.783	5.95 ± 0.73	5.37 ± 0.259	6.11 ± 0.225	5.45 ± 1.037	0.6474
Spleen	0.3 ± 0.031	0.31 ± 0.086	0.33 ± 0.031	0.29 ± 0.013	0.31 ± 0.029	0.7863
Lung	0.79 ± 0.074	0.83 ± 0.078	0.7 ± 0.178	0.68 ± 0.051	0.62 ± 0.158	0.0559
Kidney	0.74 ± 0.096	0.74 ± 0.096	0.74 ± 0.096	0.57 ± 0.037	0.55 ± 0.059	0.0184

## Data Availability

The original contributions presented in this study are included in the article. Further inquiries can be directed to the corresponding authors.
